# Life‐history differences across latitude in common side‐blotched lizards (*Uta stansburiana*)

**DOI:** 10.1002/ece3.5157

**Published:** 2019-04-18

**Authors:** Geoffrey D. Smith, Peter A. Zani, Susannah S. French

**Affiliations:** ^1^ Department of Biological Sciences Dixie State University St. George Utah; ^2^ Department of Biology University of Wisconsin‐Steven's Point Steven's Point Wisconsin; ^3^ Department of Biology Utah State University Logan Utah

**Keywords:** corticosterone, immune response, reproductive investment, survival, trade‐offs

## Abstract

1. Life‐history strategies are known to shift with latitude in many species. While life‐history variation related to body size, reproductive investment, and behavior has been studied for years, another crucial life‐history component is the immune system, which can influence an animal's survival.
2. We measured selected life‐history traits in side‐blotched lizards in southern Utah and Oregon in the field for two consecutive years and conducted a common‐garden experiment in the laboratory to determine how organisms from different latitudes optimize either immunity or reproduction. We observed lizards from southern populations, which are known to be shorter‐lived, had lower immune function during reproduction when compared to northern lizards in 2012, but the relationship reversed in the following year.
3. Our laboratory study revealed that southern lizards healed cutaneous wounds faster and had higher microbiocidal ability when compared to their northern counterparts, but lost mass doing so. The northern lizards ate more than the southern ones and maintained their body mass. It is possible that northern lizards are better adapted to taking advantage of available food resources. Alternatively, southern lizards may have exhibited sickness behavior in response to an immune challenge or reacted more strongly to the stress of captivity.
4. We found differences in life‐history strategies used by animals from different latitudes, and that these changes can shift within a population depending on the weather conditions of the year. Furthermore, when taken from the field and placed into a common‐garden environment, some of these differences in strategy appear to be intrinsic to the animals (i.e., whether they came from southern or northern populations).

## 1 INTRODUCTION

While anatomical differences across latitude have been well‐studied for many years (Allen, 1877; Bergmann, 1847), differences in life history are no less important. Because lifetime fitness guides natural selection, physiological trade‐offs between competing internal systems should be considered when evaluating latitudinal patterns. Moreau (1944) observed that birds laid fewer eggs nearer the equator compared with more temperate conspecifics, and in doing so initiated a critical discussion about life‐history theory (Ricklefs, 2000). Lack (1947) suggested that reproductive effort should be optimized by selection because of its direct fitness advantage, and that the differences in clutch size related to latitude were due to environmental limitations. However, when lifetime fitness is considered, future reproductive events can outweigh immediate reproduction if the cost of immediate reproduction is excessive (Williams, 1966). Trade‐offs must be considered.

Trade‐offs occur when biological characteristics or systems receive resources at the expense of another (Stearns, 1989). Trade‐offs are often associated with reproduction, which is a risky process that often comes at the expense of immediate health and survival (Harshman & Zera, 2007; Magnhagen, 1991; Nur, 1984). Previous studies have demonstrated that increased reproductive effort can reduce immunocompetence and subsequent survival (French & Moore, 2007; Hanssen, Folstad, & Erikstad, 2003; Nordling, Andersson, Zohari, & Lars, 1998). Conversely, increased immune activity can inhibit reproductive investment (French, Johnston, & Moore, 2007; López, Gabirot, & Martín, 2009; Råberg, Nilsson, Ilmonen, Stjernman, & Hasselquist, 2000). Furthermore, the direction of these trade‐offs is context‐dependent and can vary (a) over a species’ geographic range (Ardia, 2005), (b) with the age or potential life span of the individual (Fedorka, Zuk, & Mousseau, 2004; Kirkwood & Rose, 1991), or (c) with resource availability (French, DeNardo, & Moore, 2007). As life‐history characteristics change across a species’ range, it is important to understand how the animal's immune system responds. For instance, Martin, Pless, Svoboda, and Wikelski (2004) found that house sparrows (*Passer domesticus*) have differing responses to phytohaemagglutinin injections depending on whether the birds are from temperate or neotropical regions and whether they were in breeding or nonbreeding condition. Furthermore, animals on the edge of their range have exhibited different parasite loads compared with animals in the core of the range (Martin et al., 2017), which may affect immune function. Lee (2006) suggested that animals with higher reproductive and lower survival rates (so‐called “faster‐living” species) utilize different strategies compared to “slower‐living” species. Some studies have indicated that “slower‐living” species mount larger immune responses than “faster‐living” species (Johnson et al., 2012; Tieleman, Williams, Ricklefs, & Klasing, 2005). Thus, biologists must take these numerous factors into consideration when trying to understand life‐history trade‐offs across latitude.

In order to take such factors into account, researchers must understand not only how life‐history characteristics vary across a species’ range, but also what mediates this variation. The glucocorticoid corticosterone is thought to be one of the more important mediators of immune and reproductive trade‐offs (French, McLemore, Vernon, Johnston, & Moore, 2007), and is known to vary with latitude in some species (Eikenaar, Husak, Escallon, & Moore, 2012; Silverin, Arvidsson, & Wingfield, 1997). However, the relationship between corticosterone and the immune system is context‐dependent, making predictions difficult. For instance, corticosterone was observed to reduce the immune response to phytohaemagglutinin in temperate house sparrows (*Passer domesticus*), but not in neotropical conspecifics (Martin, Gilliam, Han, Lee, & Wikelski, 2005). The energy status of the animal is also known to affect their baseline corticosterone levels in seemingly contradictory ways. For example, Kitaysky, Kitaiskaia, Wingfield, and Piatt (2001) found caloric restriction caused increased hormone concentrations in red‐legged kittiwake chicks, but Piersma, Reneerkens, and Ramenofsky (2000) found that plasma corticosterone levels were highest in red knots when the birds were in their best body condition. These results show a complicated interplay between multiple systems and point out the difficulty in understanding these systems over large geographic areas of a species’ range.

Common side‐blotched lizards (*Uta stansburiana*) present an excellent model for studying latitudinal variation in life‐history strategies and the interplay between multiple physiological systems. These small phrynosomatid lizards span a latitudinal range from central Washington State (47°38’40” N) in the United States to southern Baja California (22°52’36”) in Mexico (Stebbins, 2003). Furthermore, Tinkle (1967) observed that side‐blotched lizards from Colorado (39° 5’35”N) had longer life spans than individuals from populations in Texas (31°51’14”N). For example, Zani and Stein (2018) reported that side‐blotched lizards can live to be seven years old in Oregon, while southern individuals rarely live longer than two years (personal observation via capture–mark–recapture studies). Likewise, in a latitudinal study encompassing 22 sites ranging from Washington state, USA (46°50’) to Sonora, Mexico (28°20’), Parker and Pianka (1975) showed that northern individuals produced fewer and larger eggs than southern individuals, indicating a shift in life‐history strategy.

Here, we seek to introduce more indicators of survival and fitness into a classic life‐history framework, namely immunity and stress reactivity, using side‐blotched lizards as study subjects. Our approach was threefold: (a) studying in a natural field context across the latitudinal range of side‐blotched lizards, (b) studying how the different latitudes might change year‐to‐year, and (c) via performing a controlled laboratory immune challenge on animals from populations originating from different latitudes. Specifically, we conducted 2 years of field studies across a ~1,000 km latitudinal gradient to measure innate immunity and immediate reproductive investment in female lizards. In addition to these field studies, we collected animals from northern and southern populations and subjected them to common‐garden experiments to determine whether the reproductive and immune differences were caused by external factors, as originally argued by Lack (1947). We hypothesized that lizards would exhibit differential investment into vital life‐history processes at different latitudes such that shorter‐lived lizards from lower latitude populations would invest more energy into immediate reproduction, while longer‐lived lizards from higher‐latitude populations would invest more energy into self‐maintenance (immunity).

## 2 MATERIALS AND METHODS

### 2.1 Animal capture and field techniques

We captured lizards via noosing at seven sites in Washington County, Utah and four sites in Harney, Lake, and Malheur Counties, Oregon in May and June 2012, totaling 91 female individuals (56 from Utah and 35 from Oregon). In May and June 2013, we sampled from six sites in Washington County, Utah and three from Harney, Lake, and Malheur Counties, Oregon, totaling 82 females (44 from Utah and 38 from Oregon). Washington County, Utah sites ranged in elevation from 775 to 1,350 m and Oregon sites ranged from 1,150 to 1,500 m. Burns, Oregon (similar in latitude and intermediate in longitude to the Oregon sites) has an average December temperature of −4°C compared to Saint George, Utah (in Washington County, where all Utah lizards were collected), which has an average December temperature of −5.2°. The Oregon sites also receive more annual precipitation (27.9 cm in Burns, Oregon) compared to the Utah sites (22.4 cm in Saint George). Furthermore, the Oregon sites receive an annual snowfall of 10.92 cm while Saint George receives very little (1.4 cm) in most years (www.ncdc.noaa.gov). To account for seasonality and its effect on phenology, we sampled Utah populations first each season to ensure we were sampling animals at approximately the same time in their reproductive cycles. Since side‐blotched lizards can lay multiple clutches of eggs each year, we attempted to sample animals during vitellogenesis of their first annual clutch to reduce differences. Upon capture, we drew blood within 3 min by rupturing the retro‐orbital sinus with a heparinized capillary tube. Then, the animals were placed into an opaque, breathable cotton bag for 10 min as a uniform stressor. After 10 min, the animals were bled again. While in the field, blood samples were maintained on ice. We kept the blood in sterile vials and separated the plasma from the pellet via centrifugation at 2,200 × *g* for 10 min. Samples were then frozen with dry ice and transported to Utah State University for assays. Considering the small size of the animals and limited blood volumes, not all individuals were used in every assay, and we prioritized the bactericidal assay above the radioimmunoassay. The lizards were measured to determine snout–vent length (SVL) with a handheld ruler to within 1 mm and weighed with a Pesola scale (Schindellegi, Switzerland) to within 0.1 g. Females were manually palpated to estimate clutch size (James & Whitford, 1994; Tinkle, 1967; Turner, Medica, & Smith, 1974), and approximate stage of vitellogenesis (firm follicles were staged early; round, soft follicles were staged mid‐vitellogenic; ovoid follicles were considered late vitellogenic; shelled eggs were staged gravid). We used a portable ultrasound unit (SonoSite, Inc., Bothell, Washington, USA) to verify clutch size and measure the lengths of each follicle. Sum follicular length was used as a measure of reproductive investment along with clutch size and was calculated by adding the greatest diameter of all follicles together for each female. Each lizard was given an individual identification number via toe‐clipping, and all animals were released at the point of capture within 24 hr.

### 2.2 Laboratory animal housing and feeding

We housed lizards from both regions in a common‐garden laboratory environment to determine whether differences observed in the field were driven by factors intrinsic or extrinsic to the organisms. Reproductively mature female lizards in early to mid‐vitellogenesis were captured via noosing in Washington County (*n* = 31), Utah and Harney County, Oregon (*n* = 22) and transported to Utah State University in May and June 2015. Lizards were housed individually in 30 × 45 × 15 cm plastic containers maintained at an ambient temperature of 23°C. The containers were lined with newspaper substrate and had a heat strip and ultraviolet light source to allow the lizards to behaviorally thermoregulate. The room was set to a photoperiod of 14L:10D and a constant relative humidity of 20%. Animals were allowed a two‐day acclimation period before being divided with a random number generator into either maintenance or restricted feeding treatments. Maintenance animals were fed three crickets (Flukers Farms, Port Allen, Louisiana, USA) daily, while restricted animals were fed three crickets every three days. No lizards were fed on the biopsy days (after 7 days of feeding), and all were given access to water ad libitum. The crickets were weighed (±0.1 g) prior to being introduced to the lizard containers and any that were not eaten by the following day were removed and weighed to quantify biomass and rates of feeding. Additionally, the lizards were weighed at the beginning and end of the study to determine mass loss or gain caused by different feeding treatments. We noted any animals that laid eggs throughout the course of this study, and no lizard reabsorbed follicles. After the experiment, Oregon animals were anesthetized following permit protocols and Utah animals were released at the capture site.

### 2.3 Cutaneous biopsy and measurements

To elicit a uniform and ecologically relevant immune challenge, we administered cutaneous biopsies to all animals of this study after 7 days of feeding treatments. Lizards were anesthetized using isoflurane (2‐chloro‐2‐(difluoromethoxy)‐1,1,1‐trifluoro‐ethane) gas. After the animals were determined to be unresponsive, a 3.5 mm circular biopsy punch (Miltex Instrument Company, York, Pennsylvania, USA) was gently twisted against the dorsal surface anterior to the base of the tail. The uniform piece of skin was then removed with forceps. Lizards were allowed to recover on heating pads until they were responsive, and they were not fed the day of the biopsies, although feeding treatments continued afterward. The wounds were photographed immediately after being administered with a ruler in the same plane as the wound for future measurements. Images were analyzed for wound area using ImageJ (NIH Imaging) such that the investigator was blind to the treatment of the animal. Wound healing rate was calculated as the percent of the wound area healed over the course of the experiment.

Following wounding, lizards were placed back into cups on heating pads and allowed to recover until they were once again alert and responsive, then returned to their containers. The wounds were photographed and analyzed again at the end of the study, 5 d later, and a single blood sample was obtained via the retro‐orbital sinus for immune and hormonal assays.

### 2.4 Radioimmunoassay

Circulating corticosterone (CORT) concentrations were determined using a radioimmunoassay protocol modified from Moore (1986). Briefly, plasma samples were extracted using a solution of 30% ethyl acetate:isooctane and assayed in duplicate for CORT (Ab: MP Biomedicals, Lot #3R3PB‐19E). Final concentrations were calculated by averaging the duplicate samples and adjusted for accuracy using individual recoveries, and all intra‐assay coefficients of variation were > 0.2.

### 2.5 Bactericidal ability

We performed bactericidal assays on samples following the protocol outlined in French and Neuman‐Lee (2012). Briefly, we combined a 1:5 plasma dilution with CO_2_‐independent media plus 4 nmol/L L‐glutamine, 10^4^ colony‐producing units of *E. coli* (EPowerTM Microorganisms #483‐237–1, ATCC 8,739, MicroBioLogics, St. Cloud, MN, USA), and agar broth on a 96‐well microplate. We also included positive (media and bacteria with no plasma) and negative (media alone) controls to account for total possible growth and ensure no contamination was present. We incubated the plate for 12 hr and calculated absorbance using a microplate reader at 300 nm (BioRad Benchmark, Hercules, CA, USA). Microbiocidal ability was calculated as 1‐(absorbance of sample/absorbance of positive controls) × 100. Positive and negative controls were present on each plate, and microbiocidal ability was calculated based on the controls for the given plate. The coefficient of variation for positive controls across all the plates in this study was 3.43%, indicating that these methods are highly repeatable.

### 2.6 Statistical analyses

Field data were compared using a two‐sample student's *t* test with unequal variance (Welch's *t* test). Comparisons in the common‐garden laboratory experiment were made using 2 × 2 factorial ANOVAs with JMP 12.0.1™ (Statistical Analysis Software, Cary, North Carolina, USA) and GraphPad Prism 8.0.1 (GraphPad Software, San Diego California USA) with significance set at *α* = 0.05. Data for corticosterone concentration and food intake were log_10_‐transformed in the common‐garden study to meet the assumptions of normality.

## 3 RESULTS

### 3.1 Field results—2012

**Figure 1:**
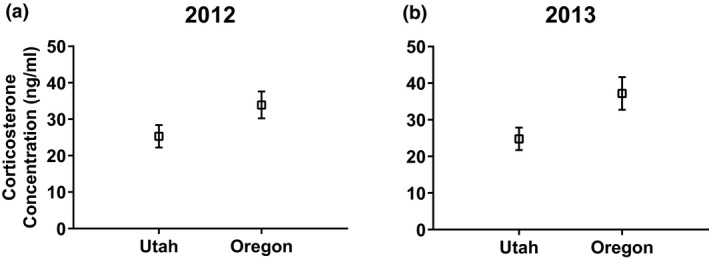
Mean (±*SE*) baseline corticosterone concentrations (ng/ml) from wild‐caught female side‐blotched lizards (*Uta stansburiana*) in (a) 2012 and (b) 2013. Lizards from northern populations (Oregon) had marginally higher (*t*(73.33) = 1.793, *p = *0.077) corticosterone concentrations compared to southern (Utah) populations in 2012, and significantly higher corticosterone concentrations (*t*(55.52) = 2.284, *p* = 0.026) in 2013

**Figure 2:**
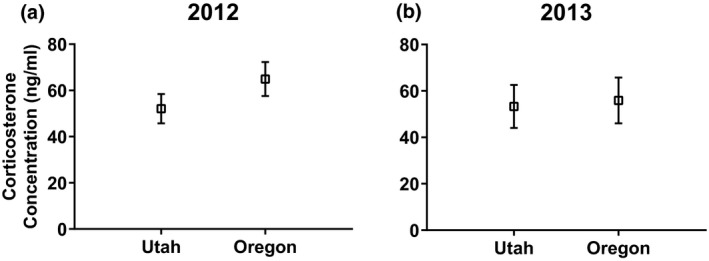
Mean (±*SE*) concentration of circulating corticosterone (ng/ml) in response to a uniform stressor in wild‐caught female side‐blotched lizards (*Uta stansburiana*) in (a) 2012 and (b) 2013. There were no significant differences in the stress response between northern and southern populations in 2012 (*t*(71.26) = 1.319, *p* = 0.192) or 2013 (*t*(58.98) = 0.190, *p* = 0.850)

**Figure 3:**
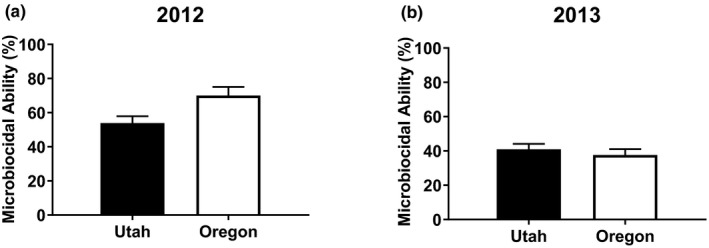
Mean (±*SE*) microbiocidal ability (%) in from wild‐caught female side‐blotched lizards (*Uta stansburiana*) in (a) 2012 and (b) 2013. Northern (Oregon) lizards had significantly higher microbiocidal capacities in 2012 compared to southern (Utah) lizards (*t*(72.54) = 2.502, *p* = 0.015), but there was no statistical difference in 2013 (*t*(78.27) = 0.703, *p* = 0.484)

**Figure 4:**
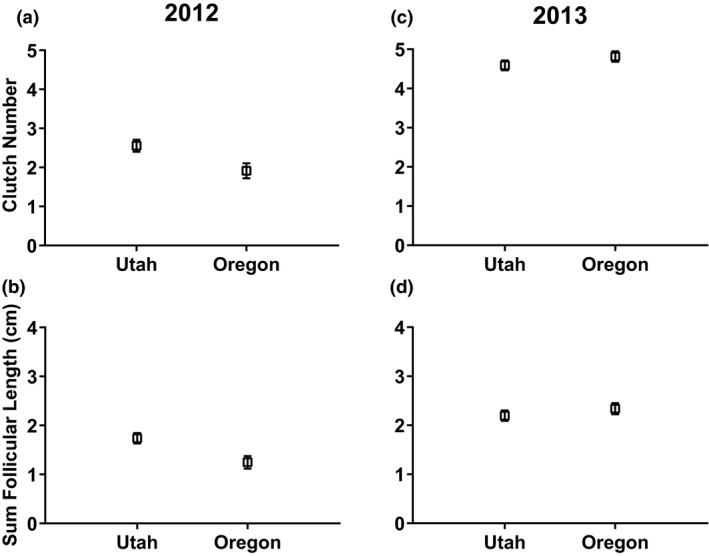
Mean (±*SE*) clutch size in 2012 (a), mean (±*SE*) sum follicular length (cm) in 2012 (b), mean (±*SE*) clutch size in 2013 (c), and mean (±*SE*) sum follicular length (cm) in 2013 (d) in wild‐caught female side‐blotched lizards (*Uta stansburiana*). Southern (Utah) lizards had significantly higher clutch sizes (*t*(72.75) = 2.604, *p* = 0.011) and sum follicular lengths (*t*(73.74) = 2.932, *p* = 0.005) in 2012 compared with northern (Oregon) lizards. There was no statistical difference in clutch size (*t*(78.27) = 1.232, *p* = 0.222) or sum follicular length (*t*(78.27) = 0.921, *p* = 0.360) in 2013

In 2012, Oregon animals had higher baseline corticosterone concentrations (33.91 ± 3.677 ng/ml, *n* = 35; mean ± *SE*, *n*) than Utah animals (25.32 ± 3.076, *n* = 50), but this difference was not significant (*t*(73.33) = 1.793, *p = *0.077) (Figure 1a). The corticosterone response was not different between regions (*t*(71.26) = 1.319, *p* = 0.192) (Figure 2a). Oregon animals had 23.07% higher microbiocidal abilities than Utah animals (*t*(72.54) = 2.502, *p* = 0.015) (Figure 3a). Utah animals had 25.39% larger clutch size (*t*(72.75) = 2.604, *p* = 0.011) at 2.56 ± 0.154 (*n* = 54) follicles compared to 1.91 ± 0.192 (*n* = 35) for Oregon animals (Figure 4a). Similarly, southern lizards also had a greater sum follicular length (*t*(73.74) = 2.932, *p* = 0.005) compared to northern lizards (1.74 ± 0.106 cm, *n* = 54 to 1.25 ± 0.130 cm, *n* = 35, respectively) (Figure 4b).

### 3.2 Field results—2013

In 2013, Oregon animals had a 33.34% higher baseline corticosterone concentrations compared to the Utah animals (*t*(55.52) = 2.284, *p* = 0.026) (Figure 1b). The corticosterone response was not different between regions, similar to 2012 (*t*(58.98) = 0.190, *p* = 0.850) (Figure 2b). In contrast to the previous year, there was no difference in microbiocidal abilities (*t*(78.27) = 0.703, *p* = 0.484) (Figure 3b). However, the microbiocidal ability was lower in 2013 than in 2012 at all sites. There was no difference in clutch size (*t*(78.27) = 1.232, *p* = 0.222) at 4.82 ± 0.134, *n* = 38 for Oregon compared to 4.59 ± 0.124, *n* = 44 for Utah (Figure 4c). Similarly, there was no difference for sum follicular length (*t*(78.27) = 0.921, *p* = 0.360) at 2.34 ± 0.114 cm, *n* = 38 for Oregon compared to 2.194 ± 0.106 cm, *n* = 44 for Utah (Figure 4d). Both measures of reproductive investment were much higher than those from the previous year.

### 3.3 Common‐garden laboratory experiment

**Figure 5:**
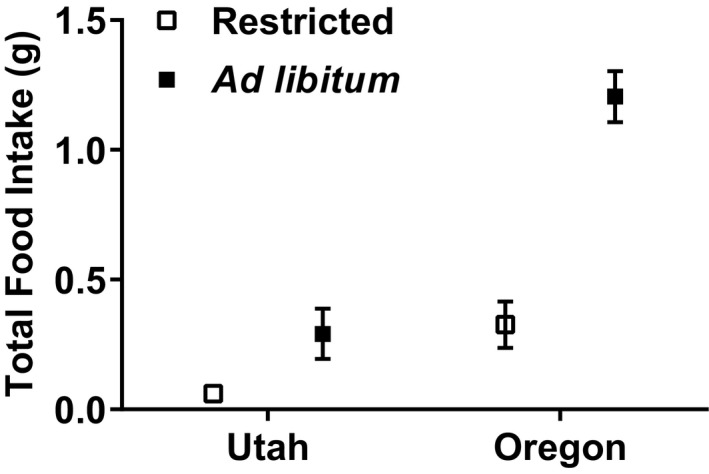
Total food intake (g) for captive female side‐blotched lizards (*Uta stansburiana*). Northern (Oregon) animals ate more than southern (Utah) animals (*F*_1,38_ = 24.735, *p* < 0.001), and maintenance animals ate more than restricted animals (*F*_1,38_ = 8.279, *p* = 0.007). There was no significant interaction between region and feeding treatment

**Figure 6:**
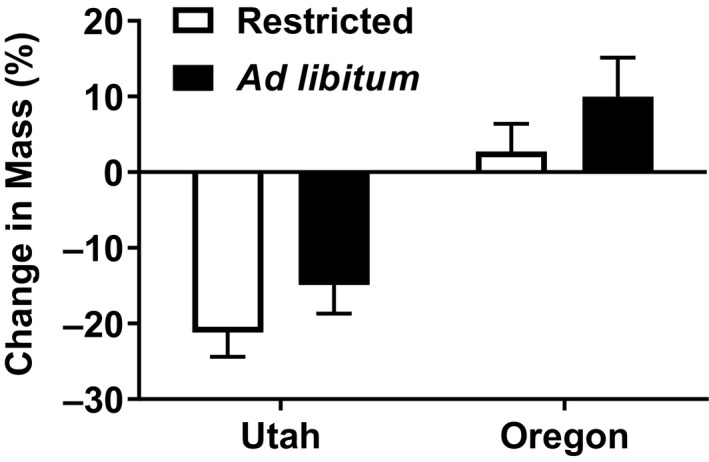
Mean (±*SE*) percent mass change for captive female side‐blotched lizards (*Uta stansburiana*). Southern (Utah) animals lost significantly more body mass than the northern (Oregon) lizards (*F*_1,48_ = 37.845, *p* < 0.001). There was no significant interaction between region and feeding treatment

**Figure 7:**
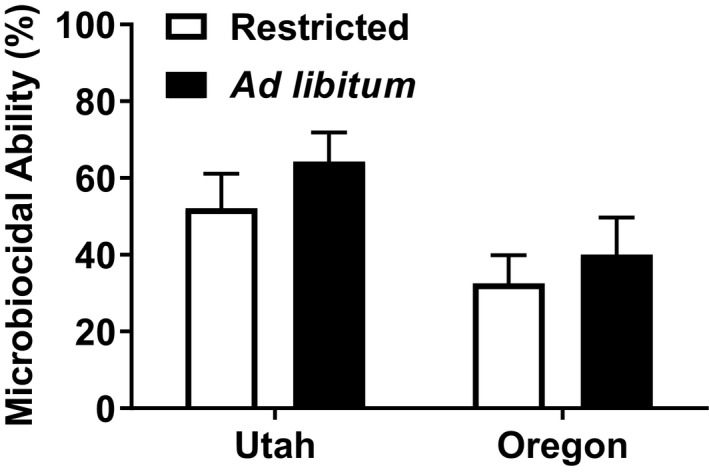
Mean (±*SE*) microbiocidal ability (%) for captive female side‐blotched lizards (*Uta stansburiana*). Southern (Utah) animals had significantly stronger microbiocidal ability than the northern (Oregon) animals (*F*_1,48_ = 6.4475, *p* = 0.014). There was no significant interaction between region and feeding treatment

**Figure 8:**
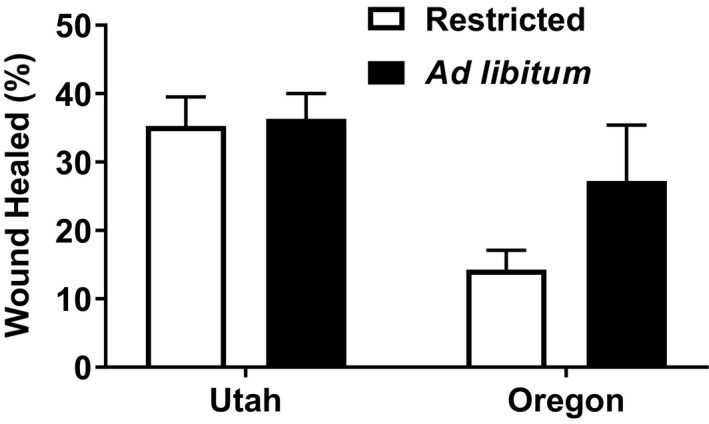
Mean (±*SE*) wound healing (%) for captive female side‐blotched lizards (*Uta stansburiana*). Southern (Utah) animals had significantly faster wound healing than the northern (Oregon) animals (*F*_1,48_ = 10.901, *p* = 0.002). There was no significant interaction between region and feeding treatment

**Figure 9:**
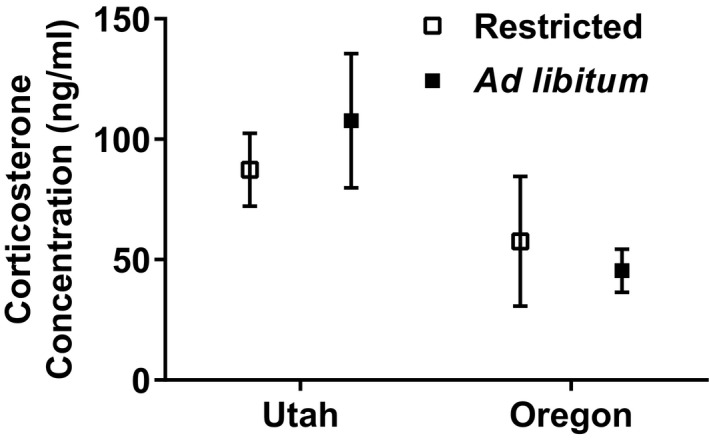
Mean (±*SE*) final corticosterone concentrations (ng/ml) for captive female side‐blotched lizards (*Uta stansburiana*). Southern (Utah) animals had significantly higher concentrations of corticosterone than northern (Oregon) lizards (*F*_1,42_ = 6.242, *p* = 0.017). There was no significant interaction between region and feeding treatment

The region of origin for lizards had a larger effect on the parameters we measured than the feeding treatments. Northern animals ate 77.53% more food than southern animals (*F*_1,38_ = 24.735, *p* < 0.001), and maintenance animals ate 75.08% more food than the restricted group (*F*_1,38_ = 8.279, *p* = 0.007) (Figure 5). There was no interaction between region of origin and feeding treatment for any dependent variable, and feeding treatment only affected the amount of food the animals ate. The southern animals lost significantly more body mass (18.04%), while the northern lizards were able to maintain their mass (*F*_1,48_ = 37.845, *p* < 0.001) (Figure 6). Southern animals exhibited 37.69% stronger microbiocidal ability than the northern lizards (*F*_1,48_ = 6.4475, *p* = 0.014) (Figure 7) and also had 45.65% faster wound healing (*F*_1,48_ = 10.901, *p* = 0.002) (Figure 8). Southern animals had 47.01% higher concentrations of corticosterone than northern lizards (*F*_1,42_ = 6.242, *p* = 0.017) (Figure 9).

## 4 DISCUSSION

The results from our 2012 field season supported our initial hypothesis that the longer‐lived lizards from higher‐latitude populations were allocating more energy into immunity at the expense of immediate reproduction. Northern animals had higher microbiocidal ability and lower clutch sizes, indicating a life‐history shift toward self‐maintenance, which is consistent with an increased likelihood of lizards surviving to a subsequent breeding season compared to the shorter‐lived southern animals. Although northern animals had 25% higher baseline concentrations of corticosterone in 2012, the difference was not statistically significant and was unlikely mediating this life‐history shift. There were no significant differences in reproductive investment or microbiocidal ability between regions in 2013 despite northern animals again having higher corticosterone concentrations.

These differences in regions and years might be explained by precipitation. In 2012 Burns, Oregon (similar in latitude and intermediate in longitude to the Oregon sites) received 1.05 cm of precipitation between January and May, but only received 0.44 cm during that interval in 2013. The 2013 precipitation in Oregon more closely resembled the precipitation received in Saint George, Utah (in Washington County, where all Utah lizards were collected) between January and May in 2012 and 2013 (0.46 and 0.58 cm, respectively) (www.ncdc.noaa.gov).

Irrespective of region, the lizards sampled in 2013 were investing in clutches in excess of four follicles, twice the averaged clutch size of 2012 animals, but consistent with previously reported clutch sizes in females from Oregon (Zani & Rollyson, 2011) and Utah (Lucas & French, 2012). Conversely, microbiocidal ability fell by more than 35% from 2012 to 2013 when regions were averaged together. It appears that southern and northern lizards alike converged on life‐history strategies in 2013, moving away from self‐maintenance and toward immediate reproduction. Interestingly, although there is a clear shift in life‐history strategy between 2012 and 2013, there is no change in corticosterone concentration, indicating that it might not be mediating these annual changes in investment.

Southern lizards had higher corticosterone at the end of the common‐garden study, indicating they might be more sensitive to stressors than their northern counterparts. However, when challenged with restraint stress in the field studies, we saw no difference with respect to region or year. It is conceivable that wound healing induced sickness behavior in these animals, which is often associated with increased corticosterone levels (Dantzer, 2001a,2001b) and reduced food intake. However, because some of the southern lizards did not feed well even before the biopsy, it is unknown whether the feeding behavior we observed was caused by the stress brought on by captivity, or if the pro‐inflammatory cytokines released as part of the immune challenge stimulated corticosterone secretion.

The immunological responses in the common‐garden experiment were also surprising. Southern animals had higher immunocompetence in two separate measures, microbiocidal ability and wound healing, despite not eating nearly as much as the northern subjects and losing more body mass. Although it is possible that the southern animals exhibited sickness behavior, whereby pro‐inflammatory cytokines were released in response to the cutaneous biopsies (Barrientos, Stojadinovic, Golinko, Brem, & Tomic‐Canic, 2008; Werner & Grose, 2003) and caused lethargy and anorexia (Johnson, 2002), the southern lizards could have been more sensitive to stress than their northern counterparts.

Additionally, animals need energy to heal wounds, but there are some situations that might make reducing foraging activity a more attractive option. Parker and Pianka (1975) hypothesized that southern populations of this species face greater predatory pressure, but other studies have found no difference in mortality due to predation (Wilson, 1991). Tail loss frequency has been used in the past as a proxy for predatory pressure (Turner, Medica, Jennrich, & Maza, 1982), but is likely an inappropriate metric due to intraspecific competition and predator efficiency (Bula, Wright, & Zani, 2015).

Regardless of how tails are lost, this injury can decrease the sprint speed of lizards (Chapple, McCoull, & Swain, 2004; Formanowicz, Daniel, Brodie, Edmund, & Bradley, 1990), making them more susceptible to predators. It is also well known that lizards reduce their home ranges (Fox & Rostker, 1982; Salvador, Martin, & López, 1995) and alter movement patterns (Martin & Avery, 1998) in response to tail loss. Cutaneous biopsies reduce sprint speed in this species similar to tail loss (S. B. Hudson, unpublished data), so it is possible that the lizards in this study were reacting to the immune challenge in behavioral ways as well as physiological. If southern animals are experiencing greater predatory or competitive pressures, they might be more likely to hide during an immune challenge than to actively forage and risk additional injury, loss of territory, or death. It is also possible that northern animals are better adapted to take advantage of ephemeral food supplies (Nussbaum, 1981), even in the context of increased external pressures such as captivity or being wounded. With a shorter period of time to acquire the resources needed to reproduce and also maintain energy reserves to survive a relatively colder winter, the northern lizards might have a higher selection pressure to obtain resources whenever they can.

We found latitudinal variation in life‐history strategy in free‐living side‐blotched lizards, but not as expected. While some characteristics of an organism might change predictably with latitude, like body size (Bergmann, 1847) and relative appendage size (Allen, 1877), other traits are less predictable. This explanation of our results is supported by our observation of converging life‐history strategies (increased reproduction at the expense of immunity) in the drought year 2013, when northern animals experienced abiotic challenges similar to the southern populations. Future studies on latitudinal variation in life history should account for the conditions the animals are facing in the current season, because strategies can change annually. However, our laboratory experiment indicates that some life‐history strategies are more deeply ingrained, with southern animals likely exhibiting sickness behavior and northern ones taking advantage of whatever food resources were available.

## CONFLICT OF INTEREST

None decalred.

## AUTHORS’ CONTRIBUTIONS

All authors contributed to the experimental design, data collection, analyses, and writing of this manuscript.

## Data Availability

Data will be archived publicly with the Dryad Digital Repository (https://doi.org/10.5061/dryad.j482vf7).

## References

[ece35157-bib-0001] Allen, J. A. (1877). The influence of physical conditions in the genesis of species. Radical Review, 1, 108–140.

[ece35157-bib-0002] Ardia, D. R. (2005). Tree swallows trade off immune function and reproductive effort differently across their range. Ecology, 86, 2040–2046. 10.1890/04-1619

[ece35157-bib-0003] Barrientos, S. , Stojadinovic, O. , Golinko, M. S. , Brem, H. , & Tomic‐Canic, M. (2008). Growth factors and cytokines in wound healing. Wound Repair and Regeneration, 16, 585–601. 19128254 10.1111/j.1524-475X.2008.00410.x

[ece35157-bib-0004] Bergmann, C. (1847). Uber die verhaltnisse der warmeokonomie der thiere zu ihrer grosse. Gottinger Studien, 1, 595–708.

[ece35157-bib-0005] Bula, P. A. , Wright, L. K. , & Zani, P. A. (2015). Geographic variation in lizard hind-limb morphology in relation to predation: No evidence for an evolutionary basis. Evolutionary Ecology Research, 16(8), 663–687.

[ece35157-bib-0006] Chapple, D. G. , McCoull, C. J. , & Swain, R. (2004). Effect of tail loss on sprint speed and growth in newborn skinks, *Niveoscincus metallicus* . Journal of Herpetology, 38, 137–140. 10.1670/128-03N

[ece35157-bib-0007] Dantzer, R. (2001a). Cytokine‐induced sickness behavior: Mechanisms and implications. Annals of the New York Academy of Sciences, 933, 222–234. 12000023 10.1111/j.1749-6632.2001.tb05827.x

[ece35157-bib-0008] Dantzer, R. (2001b). Cytokine‐induced sickness behavior: Where do we stand? Brain, Behavior, and Immunity, 15, 7–24. 10.1006/brbi.2000.0613 11259077

[ece35157-bib-0009] Eikenaar, C. , Husak, J. , Escallon, C. , & Moore, I. T. (2012). Variation in testosterone and corticosterone in amphibians and reptiles: Relationships with latitude, elevation, and breeding season length. American Naturalist, 180, 642–654. 10.1086/667891 23070324

[ece35157-bib-0010] Fedorka, K. M. , Zuk, M. , & Mousseau, T. A. (2004). Immune suppression and the cost of reproduction in the ground cricket, *Allonemobius socius* . Evolution, 58, 2478–2485. 10.1111/j.0014-3820.2004.tb00877.x 15612291

[ece35157-bib-0011] Formanowicz, J. , Daniel, R. , Brodie, J. , Edmund, D. , & Bradley, P. J. (1990). Behavioural compensation for tail loss in the ground skink, *Scincella lateralis* . Animal Behaviour, 40, 782–784. 10.1016/S0003-3472(05)80710-9

[ece35157-bib-0012] Fox, S. F. , & Rostker, M. A. (1982). Social cost of tail loss in *Uta stansburiana* . Science, 218, 692–693. 17791590 10.1126/science.218.4573.692

[ece35157-bib-0013] French, S. S. , DeNardo, D. F. , & Moore, M. C. (2007). Trade‐Offs between the reproductive and immune systems: Facultative responses to resources or obligate responses to reproduction? American Naturalist, 170, 79–89. 10.1086/518569 17853993

[ece35157-bib-0014] French, S. S. , Johnston, G. , & Moore, M. (2007). Immune activity suppresses reproduction in food in food‐limited female tree lizards *Urosaurus ornatus* . Functional Ecology, 21, 1115–1122.

[ece35157-bib-0015] French, S. S. , McLemore, R. , Vernon, B. , Johnston, G. I. , & Moore, M. C. (2007). Corticosterone modulation of reproductive and immune systems trade‐offs in female tree lizards: Long‐term corticosterone manipulations via injectable gelling material. Journal of Experimental Biology, 210, 2859–2865. 17690234 10.1242/jeb.005348

[ece35157-bib-0016] French, S. S. , & Moore, M. C. (2007). Immune function varies with reproductive stage and context in female and male tree lizards, *Urosaurus ornatus* . General and Comparative Endocrinology, 155, 148–156. 10.1016/j.ygcen.2007.04.007 17517411

[ece35157-bib-0017] French, S. S. , & Neuman‐Lee, L. A. (2012). Improved *ex vivo* method for microbiocidal activity across vertebrate species. Biology Open, 1, 482–487. 10.1242/bio.2012919 23213440 PMC3507210

[ece35157-bib-0018] Hanssen, S. A. , Folstad, I. , & Erikstad, K. E. (2003). Reduced immunocompetence and cost of reproduction in common eiders. Oecologia, 136, 457–464. 10.1007/s00442-003-1282-8 12783295

[ece35157-bib-0019] Harshman, L. G. , & Zera, A. J. (2007). The cost of reproduction: The devil in the details. Trends in Ecology & Evolution, 22, 80–86. 10.1016/j.tree.2006.10.008 17056152

[ece35157-bib-0020] James, C. D. , & Whitford, W. G. (1994). An experiemtnal study of phenotyic plasticity in the clutch size of a lizard. Oikos, 70, 49–56.

[ece35157-bib-0021] Johnson, P. T. , Rohr, J. R. , Hoverman, J. T. , Kellermanns, E. , Bowerman, J. , & Lunde, K. B. (2012). Living fast and dying of infection: Host life history drives interspecific variation in infection and disease risk. Ecology Letters, 15, 235–242. 10.1111/j.1461-0248.2011.01730.x 22221837

[ece35157-bib-0022] Johnson, R. (2002). The concept of sickness behavior: A brief chronological account of four key discoveries. Veterinary Immunology and Immunopathology, 87, 443–450. 10.1016/S0165-2427(02)00069-7 12072271

[ece35157-bib-0023] Kirkwood, T. B. , & Rose, M. R. (1991). Evolution of senescence: Late survival sacrificed for reproduction. Philosophical Transactions of the Royal Society of London B: Biological Sciences, 332, 15–24. 1677205 10.1098/rstb.1991.0028

[ece35157-bib-0024] Kitaysky, A. S. , Kitaiskaia, E. V. , Wingfield, J. C. , & Piatt, J. F. (2001). Dietary restriction causes chronic elevation of corticosterone and enhances stress response in red‐legged kittiwake chicks. Journal of Comparative Physiology B, 171, 701–709. 10.1007/s003600100230 11765979

[ece35157-bib-0025] Lack, D. (1947). The significance of clutch‐size. Ibis, 89, 302–352. 10.1111/j.1474-919X.1947.tb04155.x

[ece35157-bib-0026] Lee, K. A. (2006). Linking immune defenses and life history at the levels of the individual and the species. Integrative and Comparative Biology, 46, 1000–1015. 10.1093/icb/icl049 21672803

[ece35157-bib-0027] López, P. , Gabirot, M. , & Martín, J. (2009). Immune challenge affects sexual coloration of male Iberian wall lizards. Journal of Experimental Zoology Part A: Ecological Genetics and Physiology, 311, 96–104. 10.1002/jez.505 18942109

[ece35157-bib-0028] Lucas, L. D. , & French, S. S. (2012). Stress‐induced tradeoffs in a free‐living lizard across a variable landscape: Consequences for individuals and populations. PLoS One, 7(11), e49895. 23185478 10.1371/journal.pone.0049895 PMC3502225

[ece35157-bib-0029] Magnhagen, C. (1991). Predation risk as a cost of reproduction. Trends in Ecology & Evolution, 6, 183–186. 10.1016/0169-5347(91)90210-O 21232452

[ece35157-bib-0030] Martin, J. , & Avery, R. (1998). Effects of tail loss on the movement patterns of the lizard, *Psammodromus algirus* . Functional Ecology, 12, 794–802. 10.1046/j.1365-2435.1998.00247.x

[ece35157-bib-0031] Martin, L. B. II , Gilliam, J. , Han, P. , Lee, K. , & Wikelski, M. (2005). Corticosterone suppresses cutaneous immune function in temperate but not tropical House Sparrows, *Passer domesticus* . General and Comparative Endocrinology, 140, 126–135. 10.1016/j.ygcen.2004.10.010 15613275

[ece35157-bib-0032] Martin, L. B. , Kilvitis, H. J. , Brace, A. J. , Cooper, L. , Haussmann, M. F. , Mutati, A. , … Ardia, D. R. (2017). Costs of immunity and their role in the range expansion of the house sparrow in Kenya. Journal of Experimental Biology, 220, 2228–2235. 28404728 10.1242/jeb.154716

[ece35157-bib-0033] Martin, L. B. II , Pless, M. , Svoboda, J. , & Wikelski, M. (2004). Immune activity in temperate and tropical house sparrows: A common‐garden experiment. Ecology, 85, 2323–2331. 10.1890/03-0365

[ece35157-bib-0034] Moore, M. C. (1986). Elevated testosterone levels during nonbreeding‐season territoriality in a fall‐breeding lizard, *Sceloporus jarrovi* . Journal of Comparative Physiology A, 158, 159–163. 10.1007/BF01338559 3723433

[ece35157-bib-0035] Moreau, R. E. (1944). Clutch‐size: A comparative study, with special reference to African birds. Ibis, 86, 286–347. 10.1111/j.1474-919X.1944.tb04093.x

[ece35157-bib-0036] Nordling, D. , Andersson, M. , Zohari, S. , & Lars, G. (1998). Reproductive effort reduces specific immune response and parasite resistance. Proceedings of the Royal Society of London. Series B: Biological Sciences, 265(1403), 1291–1298. 10.1098/rspb.1998.0432.

[ece35157-bib-0037] Nur, N. (1984). The consequences of brood size for breeding blue tits I. Adult survival, weight change and the cost of reproduction. The Journal of Animal Ecology, 53, 479–496.

[ece35157-bib-0038] Nussbaum, R. A. (1981). Seasonal shifts in clutch size and egg size in the side‐blotched lizard, *Uta stansburiana* Baird and Girard. Oecologia, 49, 8–13. 10.1007/BF00376891 28309442

[ece35157-bib-0039] Parker, W. S. , & Pianka, E. R. (1975). Comparative ecology of populations of the lizard *Uta stansburiana* . Copeia, 1975, 615–632. 10.2307/1443314

[ece35157-bib-0040] Piersma, T. , Reneerkens, J. , & Ramenofsky, M. (2000). Baseline corticosterone peaks in shorebirds with maximal energy stores for migration: A general preparatory mechanism for rapid behavioral and metabolic transitions? General and Comparative Endocrinology, 120, 118–126. 11042017 10.1006/gcen.2000.7543

[ece35157-bib-0041] Råberg, L. , Nilsson, J. , Ilmonen, P. , Stjernman, M. , & Hasselquist, D. (2000). The cost of an immune response: Vaccination reduces parental effort. Ecology Letters, 3, 382–386. 10.1046/j.1461-0248.2000.00154.x

[ece35157-bib-0042] Ricklefs, R. E. (2000). Lack, Skutch, and Moreau: The early development of life‐history thinking. The Condor, 102, 3–8. 10.1650/0010-5422(2000)102[0003:LSAMTE]2.0.CO;2

[ece35157-bib-0043] Salvador, A. , Martin, J. , & López, P. (1995). Tail loss reduces home range size and access to females in male lizards, *Psammodromus algirus* . Behavioral Ecology, 6, 382–387.

[ece35157-bib-0044] Silverin, B. , Arvidsson, B. , & Wingfield, J. (1997). The adrenocortical responses to stress in breeding willow warblers *Phylloscopus trochilus* in Sweden: Effects of latitude and gender. Functional Ecology, 11, 376–384. 10.1046/j.1365-2435.1997.00097.x

[ece35157-bib-0045] Stearns, S. C. (1989). Trade‐offs in life‐history evolution. Functional Ecology, 3, 259–268. 10.2307/2389364

[ece35157-bib-0046] Stebbins, R. C. (2003). A field guide to western reptiles and amphibians. New York, NY: Houghton Mifflin.

[ece35157-bib-0047] Tieleman, B. I. , Williams, J. B. , Ricklefs, R. E. , & Klasing, K. C. (2005). Constitutive innate immunity is a component of the pace‐of‐life syndrome in tropical birds. Proceedings of the Royal Society of London B: Biological Sciences, 272, 1715–1720. 10.1098/rspb.2005.3155. PMC1559858 16087427

[ece35157-bib-0048] Tinkle, D. W. (1967). The life and demography of the side‐blotched lizard, *Uta stansburiana* . Miscellaneous Publications of the Museum of Zoology, University of Michigan, 132, 1–182.

[ece35157-bib-0049] Turner, F. B. , Medica, P. A. , Jennrich, R. I. , & Maza, B. G. (1982). Frequencies of broken tails among *Uta stansburiana* in southern Nevada and a test of the predation hypothesis. Copeia, 4, 835–840. 10.2307/1444094.

[ece35157-bib-0050] Turner, F. B. , Medica, P. A. , & Smith, D. D. (1974). Reproduction and survivorship of the lizard, Uta stansburiana, and the effects of winter rainfall, density and predation on those processes. U.S. International Biological Program, Desert Biome, Utah State University, Logan, Utah, Reports of 1973 Progress, 3, 117–128.

[ece35157-bib-0051] Werner, S. , & Grose, R. (2003). Regulation of wound healing by growth factors and cytokines. Physiological Reviews, 83, 835–870. 10.1152/physrev.2003.83.3.835 12843410

[ece35157-bib-0052] Williams, G. C. (1966). Adaptation and natural selection: A critique of some current evolutionary thought (p. 307). Princeton, NJ: Princeton University Press.

[ece35157-bib-0053] Wilson, B. S. (1991). Latitudinal variation in activity season mortality rates of the lizard *Uta stansburiana* . Ecological Monographs, 61(4), 393–414.

[ece35157-bib-0054] Zani, P. A. , & Rollyson, M. E. (2011). The effects of climate modes on growing‐season length and timing of reproduction in the Pacific Northwest as revealed by biophysical modeling of lizards. American Midland Naturalist, 165, 372–388. 10.1674/0003-0031-165.2.372

[ece35157-bib-0055] Zani, P. A. , & Stein, S. J. (2018). Field and laboratory responses to drought by common side‐blotched lizards (*Uta stansburiana*). Journal of Arid Environments., 154, 15–23. 10.1016/j.jaridenv.2018.03.001

